# Characterization of Stra8 in Southern catfish (*Silurus meridionalis*): evidence for its role in meiotic initiation

**DOI:** 10.1186/1471-2199-14-11

**Published:** 2013-05-22

**Authors:** Ranran Dong, Shijie Yang, Jing Jiao, Tingru Wang, Hongjuan Shi, Linyan Zhou, Yaoguang Zhang, Deshou Wang

**Affiliations:** 1 Key Laboratory of Freshwater Fish Reproduction and Development (Ministry of Education), Key Laboratory of Aquatic Science of Chongqing, School of Life Science, Southwest University, Chongqing, China

**Keywords:** *Stra8*, mRNA and protein, Expression profile, Meiotic initiation, *Silurus meridionalis*

## Abstract

**Background:**

RA (retinoic acid) signal pathway has been proved to be required for germ cell meiotic initiation in mammals, aves and amphibians. Stra8 (Stimulated by retinoic acid gene 8) is an important factor in RA signal pathway. However, the role of RA and Stra8 in germ cell meiotic initiation in teleosts is poorly characterized.

**Results:**

In this study, the full length cDNA of *Stra8* was cloned from Southern catfish (*Silurus meridionalis*), and its spatio-temporal expression profiles were analyzed. The *Stra8* cDNA (1606 bp) includes 163 bp 5’-UTR (untranslated region), 456 bp 3’-UTR, and an ORF (open reading frame) of 987 bp, encoding a polypeptide of 328 aa. Phylogenetic analysis revealed its existence in some primitive teleosts, such as Siluriformes and Salmoniformes. Tissue distribution analysis by RT-PCR showed that *Stra8* is specifically expressed in gonads. By real-time PCR, *in situ* hybridization and immunohistochemistry, the highest expression level of *Stra8/*Stra8 was detected in 50 and 130 dah (day after hatching), the premeiotic stage of germ cells in XX and XY gonads, respectively.

**Conclusions:**

Our results suggest that Stra8 might be involved in germ cell meiotic initiation in *S. meridionalis* as it did in tetrapods.

## Background

Meiosis is a special type of cell division necessary for sexual reproduction in eukaryotes. It not only generates genetic diversity on which natural selection can act, but also helps maintain the immortality of the germ line [1]. In mammals, meiosis is initiated at different time points in males and females. Recent discoveries indicate that the key to this sex-specific timing of meiosis entry is the presence or absence of the signaling molecule RA (retinoic acid) [2-7]. In mouse, a model was proposed in which RA signaling and metabolism regulate whether female and male germ cells initiate meiosis during embryogenesis. The model posits that, in embryonic ovaries, RA induces germ cells to express Stra8, which in turn leads to meiotic initiation. In embryonic testes, an enzyme of the Cyp26 family, likely Cyp26b1, degrades RA and thereby prevents expression of Stra8 and precludes meiotic initiation [8-10]. In avians and amphibians, the role of RA in meiotic initiation is similar to that in mammals showing that RA-dependent meiosis entry could be a conserved mechanism of germ cell differentiation in tetrapods [11,12].

Stra8 which was first identified in a screen for genes induced by RA is specifically expressed in mammalian germ cells before their transition from mitosis to meiosis [13-16]. Studies on the teratocarcinoma cells and embryonic stem cells (ESC) transfected with Stra8-EGFP shown that the positive cells may undergo meiosis develop into sperm and generate live offspring mice [16]. In female embryos lacking *Stra8* gene function, the early mitotic development of germ cells is normal, but these cells then fail to undergo premeiotic DNA replication, meiotic chromosome condensation, cohesion, synapsis and recombination [17]. Analysis of Stra8-deficient testes of mice demonstrated that Stra8 is essential for normal progression into meiotic prophase [4,14,17]. Above all, Stra8 is the key factor involved in RA signal pathway in the control of germ cell meiosis.

The role of Stra8 in the meiosis of teleost has not yet been reported. Some researchers even suspected that Stra8 may be amniote- or even mammalian-specific, according to Ensemble orthologue prediction (http://www.ensembl.org/index.html) [18]. In the present study, homologous sequences of *Stra8* were isolated from channel catfish, rainbow trout and salmon in EST database, and *Stra8* cDNA was cloned from *S. meridionalis*. The spatial and temporal expression patterns of *Stra8* were examined by RT-PCR, real-time PCR, *in situ* hybridization and immunohistochemistry. The study shows for the first time that *Stra8* is expressed highest in the premeiotic phase of germ cell in *S. meridionalis*. Our data indicated that Stra8 may be involved in meiotic initiation of *S. meridionalis*.

## Results

### Cloning and characterization of *S. meridionalis Stra8* cDNA

Using degenerated PCR amplification, a cDNA fragment of 642 bp was obtained and was confirmed to be a partial sequence of *S. meridionalis Stra8* gene by homology search in the GenBank database. The full length cDNA of *Stra8* was successfully isolated by RACE-PCR. *Stra8* cDNA (accession number: KC533813) was 1606 bp in length containing an open reading frame (ORF) of 987 bp, corresponding to 328 amino acid residues and the 5’ and 3’ UTRs of 163 and 483 bp, respectively. The predicted molecular mass and pI value of the deduced Stra8 protein were 43 kDa and 4.29, respectively.

### Amino acid sequence alignment

**Figure 1:**
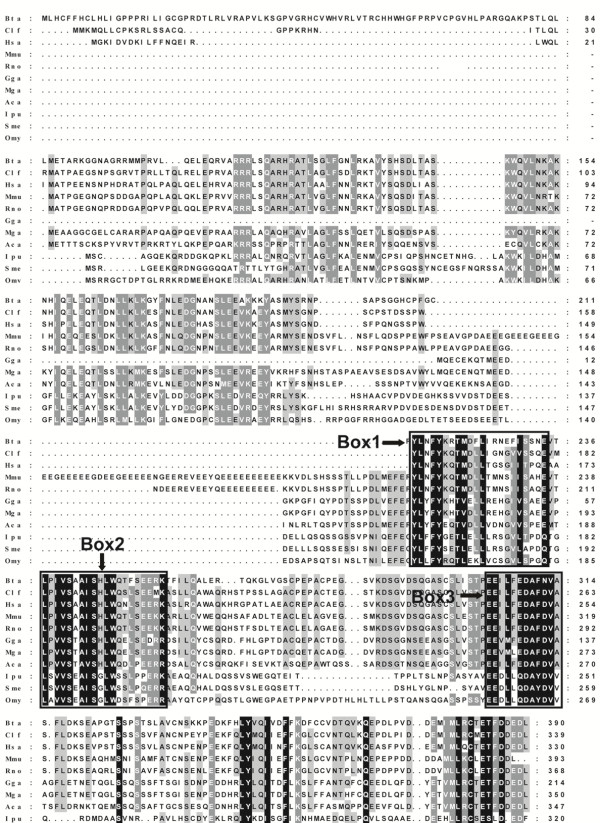
**Alignments of *S. meridionalis *Stra8 with those of other species.** ClustalX and GeneDoc were used to make this figure. The overall similarity among the isolated Stra8 is very low, averagely less than 30%. The three relatively conserved motifs were depicted as Box 1–3. The sources of the sequences are described in the Materials and Methods section. *Has*, *H.sapiens*; *Bta*, *B. taurus*; *Mmu*, *M. musculus*; *Rno*, *R. norvegicus*; *Gga*, *G. gallus*; *Clf*, *C. lupus familiaris*; *Omy*, *O. mykiss*; *Aca*, *A. carolinensis*; *Mga*, *M. gallopavo*; *Ipu*, *I. punetaus*; *Sme*, *S. meridionalis.*

Alignment of the *S. meridionalis* Stra8 with those from other organisms revealed a low identity, especially in the N-terminal regions (Figure 1). The overall similarity between fish and tetrapods Stra8 is very low, averagely less than 30%. It shared 28% similarity with *H. sapiens* (NP_872295), 29% with *M. musculus* (NP_033318) and *M. gallopavo* (XP_003202232), 30% with *G. gallus* (XP_416179), 27% with *A. carolinensis* (XP_003221102), and 23% with *X. tropicalis* (XP_002941476). However, *S. meridionalis* Stra8 shared 75% with *I. punctatus*, and 44% with *O. mykiss*, respectively. There are three motifs (depicted as Box 1–3) relatively conserved among all Stra8. Box3 is the most important and conserved signature motif. All fish share the same 12 amino acid sequences in this motif while only 3 out of 12 amino acid residues differ between Stra8 from fish and tetrapods.

### Phylogenetic analysis of Stra8

**Figure 2:**
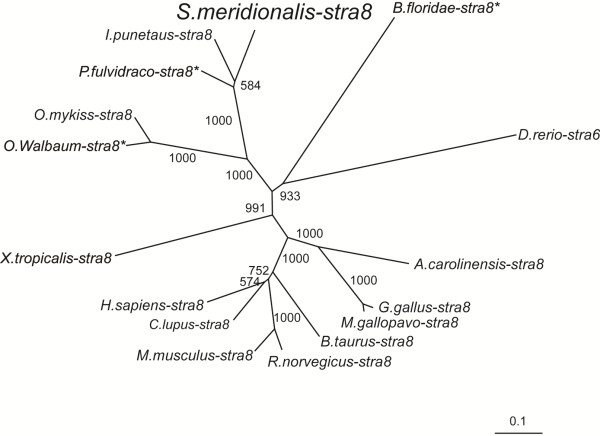
**Phylogenetic tree of Stra8.** The tree was made with the default settings of the ClustalX protein alignment program and visualized using treeview32. The values represent bootstrap scores out of 1000 trials, indicating the credibility of each branch. Asterisks indicate partial sequences.

Phylogenetic analysis of Stra8 from representative cephalochordate, fish, amphibians, reptiles, avians and mammals (Figure 2) produced an NJ-phylogenetic tree that contained three distinct branches. The *S. meridionalis* Stra8 clustered with those of fish, while sequences from tetrapod and cephalochordate formed a separate cluster, respectively.

### Tissue distribution by RT-PCR

**Figure 3:**
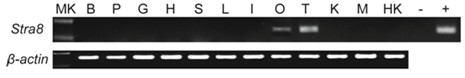
**RT-PCR analysis of Stra8 from various adult *S.meridionalis *tissues.** B, brain; P, pituitary; G, gill; H, heart; S, spleen; L, liver; I, intestine; O, ovary; T, testis; K, kidney; M, muscle; HK, head kidney; +, positive control; -, negative control. Lower panel: β-actin was used as internal control.

In adult *S. meridionalis*, *Stra8* was found to be exclusively expressed in gonads, with much higher expression in the testis compared with the ovary (Figure 3).

### Expression profiles of *Stra8* in gonads by real-time PCR

**Figure 4:**
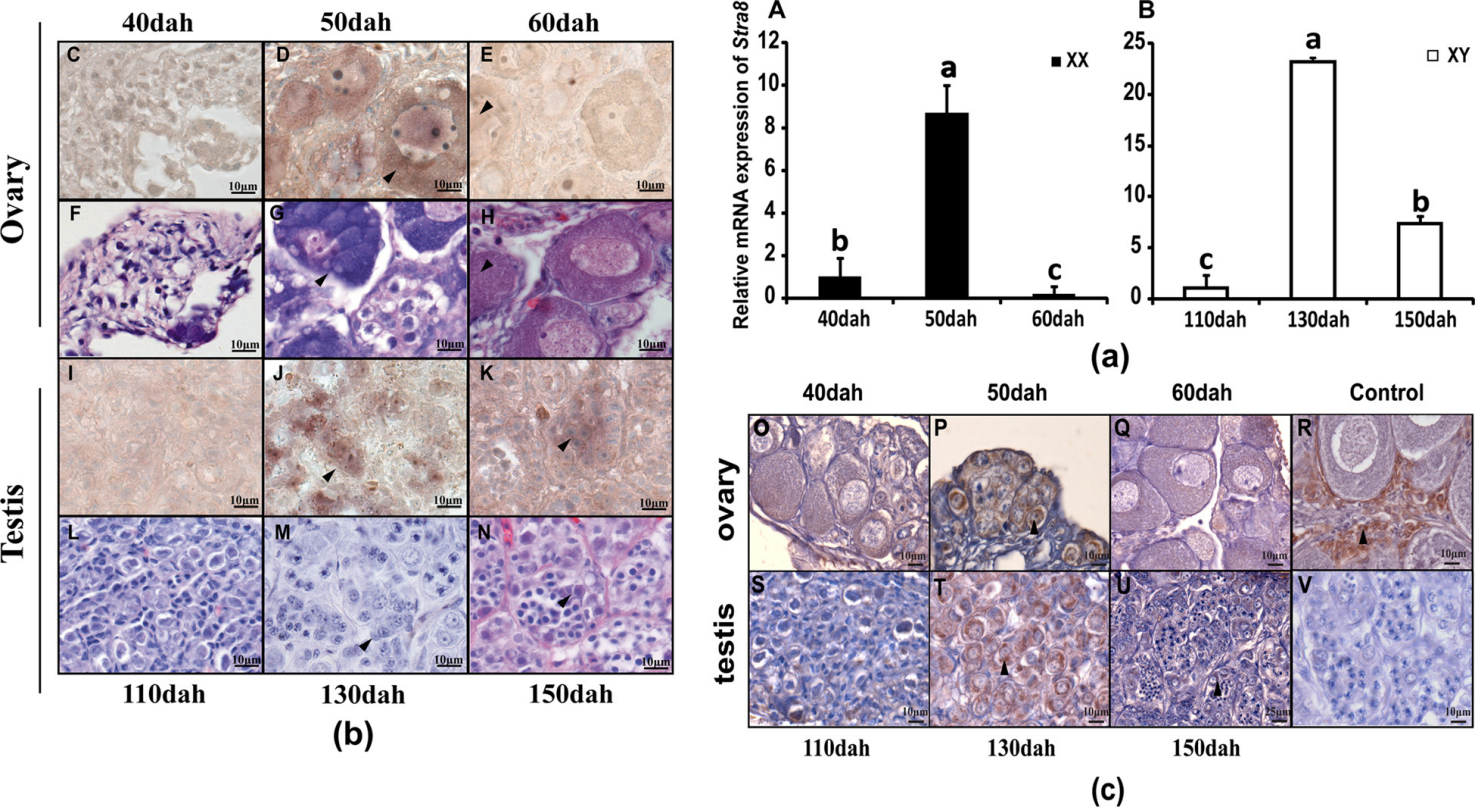
**Expression profile of *Stra8*/Stra8 in *S. meridionalis *gonads during germ cell meiotic initiation analyzed by real-time PCR (a), hemotoxylin-eosin (H.E.) staining (b), in situ hybridization and immunohistochemistry (c), respectively.** (**a**), Values represent the relative mRNA expression. Data were expressed as the mean ± SE of the triplicates. Bars bearing the same letters are not significantly different at P < 0.01 by one-way ANOVA. Weak mRNA and protein signals was detected in 40 dah and 60 dah ovaries and 110 dah and 150 dah testes (**C**, **E**, **I**, **K**, **O**, **Q**, **R** and **T**). Strong signals were found in premeiotic germ cells in 50 dah ovaries and 130 dah testes (**D**, **J**, **P** and **S**). The positive signal corresponds to the brownish color (**R**). Arrowhead indicates the signals of *Stra8*/Stra8 in ovaries and testes, respectively. H.E. staining (**F**-**H**, **L**-**N**).

As shown in Figure 4(a), the relative expression of *Stra8* mRNA was at the highest level in 50 dah ovaries and 130 dah testes, corresponding to the premeiotic stage of germ cells, and at lower levels in 40 and 60 dah ovaries as well 110 and 150 dah testes.

### Localization of Stra8 in gonads

#### Morphological observation of gonads by H.E. staining

To observe the germ cell meiosis in female and male gonads, H.E. staining was performed using ovaries at 40, 50 and 60 dah and testes at 110, 130 and 150 dah. The results showed that ovaries at 40, 50 and 60 dah were mainly composed of premeiotic oogonia and primary oocytes, respectively; while testes at 110, 130 and 150 dah, were basically composed of synchronized spermatogonia and primary spermatocytes (Figure 4 b). The results also showed that the majority germ cells are in the premeiotic phase in 50 dah ovaries and testes130 dah.

#### Expression, purification, and Western blot analysis

**Figure 5:**
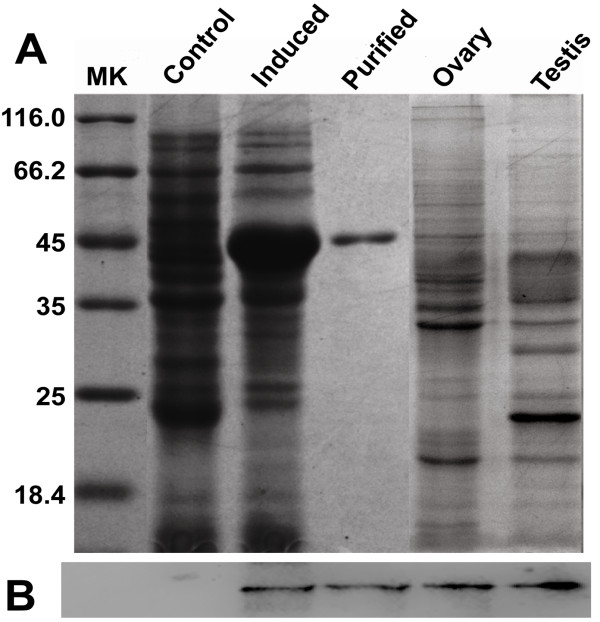
**SDS-PAGE and Western blot analyses of recombinant His-Stra8 proteins and total proteins extracted from*S. meridionalis *gonads. a**) Stra8 protein with His-tag expressed in *E. coli* and total proteins extracted from 50 dah ovaries and 130 dah testes of *S. meridionalis* were analyzed by SDS-PAGE followed by Coomassie blue staining. **b**) The endogenous mature peptide of Stra8 was further demonstrated with Stra8 polyclonal antibody by chemiluminescence.

Recombinant Stra8 protein with a His-tag at its N terminus was successfully expressed in *E. coli*. Unpurified and purified recombinant Stra8 proteins were analyzed by SDS-PAGE with Coomassie blue staining (Figure 5). The specificity of the Stra8 antibody was confirmed by Western blotting. Specific bands of 43 kDa corresponding to the calculated molecular weights of the *S. meridionalis* Stra8 fusion proteins and total proteins extracted from 50 dah ovaries and 130 dah testes were recognized using our own Stra8 antibody (Figure 5).

#### Cell types of *Stra8*/Stra8 in gonads by ISH and IHC

To identify the localization of *Stra8*/Stra8 in gonads, ISH and IHC was performed using ovaries at 40, 50 and 60 dah and testes at 110, 130 and 150 dah. The results showed that specific signals of *Stra8*/Stra8 were observed in germ cells in gonads of both sexes. Weak mRNA and protein signals were detected in 40 dah ovaries and 110 dah testes (Figure 4 C, I, O and S). Strong signals were found in premeiotic germ cells in 50 dah ovaries and 130 dah testes (Figure 4 D, J, P and T). The signals became weaker in the 60 dah ovary and 150 dah testes (Figure 4 E, K, Q and U). The positive signal corresponds to the brownish color (Figure 4 R). No signal was detected in the negative control without the primary antibody (Figure 4 V).

## Discussion

In this study, the full length cDNA of the *Stra8* was cloned and characterized in a teleost, *S. meridionalis*, for the first time. Stra8 sequences were found to be poorly conserved among vertebrates. According to the phylogenetic tree, Stra8 might be existed in the common ancestor of cephalochordate and vertebrate. It is speculated that like in tetrapods Stra8 may be widespread in teleosts. However, in teleosts, *Stra8* was not found in the genome databases of zebrafish, medaka, fugu, tetraodon, stickleback and Nile tilapia. *Stra8* sequences were only isolated from Siluriformes and Salmoniformes by bioinformatics analysis of available databases. There are two possibilities for this, 1) It is difficult to identify *Stra8* from other fish species because of the very low sequence similarity; 2) *Stra8* is not included in the databases because of incomplete genome sequencing.

In mammals, *Stra8* was expressed in embryonic ovaries just before meiotic initiation, whereas its expression in testes was first detected after birth [2]. In this study, tissue distribution analysis showed that *Stra8* was specifically expressed in gonads of both sexes of adult *S. meridionalis*. In mouse, moderate levels of *Stra8* transcript in gonocytes and the peak of *Stra8* mRNA expression coincided with the onset of meiosis in postnatal testes. *Stra8* mRNA levels were greatly reduced or absent in germ cells once they entered meiosis by microarrays [19]. Interestingly, in this study real-time PCR showed that *Stra8* was expressed at the highest level in ovaries at 50 dah and testes at 130 dah. In addition, our previous work showed that meiosis of germ cell in *S. meridionalis* initiated at about 50 dah, whereas meiosis of male germ cells initiated at about 130 dah [20]. This unique expression pattern made us curious to examine the cellular localizations of *Stra8* transcripts and protein in gonads. ISH and IHC analysis revealed that *Stra8*/Stra8 signals were only observed in germ cells. Consistant with the Real-time PCR results, strongest signals were detected in ovaries at 50 dah and testes at 130 dah, respectively. Furthermore, hemotoxylin-eosin (H.E.) staining verified that the majority of germ cells in 50 dah ovaries and in 130 dah testes were in the premeiotic phase. These results suggest that Stra8 may play an important role in meiotic initiation in *S. meridionalis*.

It is well document that meiosis is initiated through retinoic acid induction of Stra8 in both male and female germ lines in higher vertebrates [21-25]. There is also evidence for similar role of RA in germ cell meiotic initiation in amphibians [12]. In addition, Cyp26b1, an RA catabolic enzyme, was proved to regulate germ cell fate in a manner common among vertebrates in Japanese flounder [26]. Long term treatment of XX Nile tilapia with Citral, an RA synthetase inhibitor, from 5 dah resulted in female to male sex reversal (unpublished data from our group). In the present study, we showed evidence for possible involvement of Stra8, the key factor for RA signal pathway, in germ cell meiotic initiation in *S. meridionalis*. Taken together, these data strongly suggest that RA may play important roles in germ cell meiotic initiation in teleosts as it did in tetrapods.

## Conclusions

*Stra8* cDNA full-length was successfully cloned from *S. meridionalis*. Sequence and phylogenetic analyses demonstrated that it is the genuine counterpart of mammalian Stra8. Tissue distribution analysis revealed that it was exclusively expressed in the gonad. Real-time PCR, ISH and IHC analyses showed that it was highly expressed in the premeiotic phase of germ cells in both ovaries and testes. These results indicated that Stra8 probably participated in meiotic initiation in *S. meridionalis* as it did in higher vertebrates. Stra8 is the key factor in RA induced germ cell meiotic initiation. Together with the results from Japanese flounder and tetrapods, we conclude that RA-dependent meiotic initiation might be a conserved mechanism of germ cell differentiation in vertebrates.

## Methods

### Animals

Adult *S. meridionalis* which has XY heterogametic sex determination were obtained from the Jialing River, a branch of the Yangtze River, and were kept in aerated tanks until use. All fry used in the experiments were obtained by artificial propagation using the same parental fish raised in our laboratory. All animal experiments conformed to the Guide for Care and Use of Laboratory Animals and were approved by the Committee of Laboratory Animal Experimentation at Southwest University, Chongqing, China.

The pre-meiotic, meiotic and post-meiotic stages of germ cells were assessed according to meiotic initiation time and germ cell morphology characterized in *S*. *meridionalis*[20]. Gonads were sampled at these stages for real-time PCR, H.E., ISH and ICH staining.

### Nucleic acid preparation and first-strand cDNA synthesis

Total RNA was extracted from various tissues using Tissue RNA Rapid Extraction Kit (TaKaRa, Japan) according to the manufacturer's protocol. The quantity and quality of RNA were examined by UV-spectrophotometry (OD260/OD280) and agarose gel electrophoresis, respectively. Total RNA (500 ng) from various tissues were reverse transcribed into first-strand cDNA using PrimeScript RT Master Mix Perfect Real Time Kit (TaKaRa, Japan) according to the manufacturer's instructions.

### Degenerated RT-PCR (reverse transcription polymerase chain reaction)

Homologous sequences of *Stra8* were isolated from Channel Catfish, rainbow trout and salmon in EST database. However, *Stra8* was not found in the genome databases of zebrafish, medaka, fugu, tetraodon, stickleback and Nile tilapia. Degenerated primers (Table 1) were designed based on the conserved domain from known *Stra8* homology sequences and were used to amplify the target fragment of the *Stra8* cDNA. First strand cDNA was subjected to PCR amplification using Premix Ex Taq™Hot Start Version (TaKaRa, Japan) and degenerated primers (10 μM) were used at final concentrations in a 25 μl reaction for 94°C for 3 min, followed by 30 cycles of 94°C for 30 s, 58°C for 30 s, 72°C for 2 min and a final elongation step at 72°C for 8 min. PCR products were separated on 1.5% agarose gel and purified with an Gel Extraction Kit (Omiga BioTek, USA). The purified PCR products were cloned into a pMD19-T vector (TaKaRa, Japan) overnight. The recombinant plasmid was transformed into *E. coli* competent cells DH5α and positive clones were sequenced at Life Technologies Corporation (Shanghai, China). The retrieved sequences were verified and analyzed for similarity with other known *Stra8* sequences using the BLASTX program at the National Center for Biotechnology Information (http://www.ncbi.nlm.nih.gov/blast.cgi).

**Table 1:** Primers used in the present study

**Primer name**	**Primer sequence (5’ to 3’)**	**Purpose**
*Stra8*-F_1_	CCAACATCCAGGAGTTTGA	cDNA fragment PCR
*Stra8*-F_2_	GGGATATCTCTTTTTCTAC	
*Stra8*-F_3_	GCCATCTCAGGCCTGTGG	
*Stra8*-R_3_	CCTGAGATGGCCTCGGACACTAC	
*Stra8*-R_2_	CTGTAGTAGATCCTCCTCA	
*Stra8*-R_2_	CACCACATCATAGGCATCCTG	
*Stra8*-RACE-F_1_	GAGCTTCTCTTGCGCAGTGGAGTG	RACE PCR
*Stra8*-RACE-F_2_	GTCCGAGGCCATCTCAGGCCTGTGGA	
*Stra8*-RACE-F_3_	CCACTGACTCACATCTCTACGGCTT	
*Stra8*-RACE-R_3_	CACTCCACTGCGCAAGAGAAGCTCGA	
*Stra8*-RACE-R_2_	CACAGGCCTGAGATGGCCTCGGACACT	
*Stra8*-RACE-R_1_	GCCGTAGAGATGTGAGTCAGTGGTCT	
*Stra8*-sF_0_	CCTCAGCAAGCTCCTTGCACTGAA	Tissue distribution
*Stra8*-sR_0_	GCTCTGCAGTACTGAAGGCCTGTT	
*Stra8*-F_0_	GAGCAGGTATGTCCCGTTTGG	Full length cDNA amplification
*Stra8*-R_0_	GGAGGAACTGGCTAGAAATCC	
*Stra8*-Q-F	AGTGCCTGATGTAGATGA	Real-time PCR
*Stra8*-Q-R	AGAGTCTCGCTGTAGAAG	
sc-β-actin-F	GGCATCACACCTTCTACAACGA	Internal control
sc-β-actin-R	ACGCTCTGTCAGGATCTTCA	

### Rapid amplification of cDNA ends (RACE)

Full-length *Stra8* cDNA was obtained by 5’- and 3’-RACE method using the SMARTer™RACE cDNA Amplification Kit (Clontech, USA) according to the manufacturer's protocol. The 5’- and 3’-RACE primers (Table 1) were designed based on the obtained *Stra8* cDNA fragment sequences. The PCR program was performed for 26 cycles of 94°C for 30 s, 68°C for 30 s and 72°C for 3 min. The amplified cDNA fragments were cloned and sequenced as described for degenerated RT-PCR above. Then the full-length *Stra8* cDNA was sequenced again to confirm the nucleotide sequences.

### Multiple sequence alignment and phylogenetic analysis

Deduced protein sequences of Stra8 from various species were retrieved from GenBank and phylogenetically compared with those of *S*. *meridionalis*. Multiple alignments were carried out using ClustalX and GeneDoc [27]. A bootstrapped neighbor-joining phylogenetic tree using *D. rerio* Stra6 (NP_001038777) as outgroup was constructed and viewed with Treeview 32. The credibility of the branching was tested using bootstrap resampling with 1000 pseudo replicates. Except *S. meridionalis* Stra8 which was cloned in this study, all other Stra8 protein sequences were obtained from the NCBI database (http://www.ncbi.nlm.nih.gov/). The accession numbers of these Stra8 protein sequences are: *H. sapiens* (NP_872295), *C. familiaris* (XP_852820), *M. musculus* (NP_033318), *R. norvegicus* (XP_575429), *B. Taurus* (XP_001253650), *M. gallopavo* (XP_003202232), *G. gallus* (XP_416179), *A. carolinensis* (XP_003221102), *X. tropicalis* (XP_002941476), *O. mykiss* (CX037430), *S. salar* (DW470925).

### Tissue distribution by RT-PCR

Gene-specific primers were used for the RT-PCR analysis. Positive and negative controls were set up with plasmid DNA and water, respectively, as templates to validate the distribution pattern. A 342 bp fragment of *β-actin* was amplified (as internal control) from *S. meridionalis* to test the quality of the cDNAs used in the PCR. The PCR conditions consisted of 94°C (3 min), followed by 30 cycles of 94°C (30s), 62°C (30s), and 72°C (30s), the reaction was ended by a further 10 min at 72°C. PCR was performed on a C1000 thermal cycler (Bio-Rad, USA). All the PCR products were subjected to agarose gel (1.5%) electrophoresis and gels were stained with ethidium bromide to visualize bands.

### Real-time PCR analysis

mRNA expression analysis for different meiotic stages of ovaries and testes were conducted via Real-time PCR according to the following method: gene-specific primers (Table 1) were designed based on the cloned *Stra8* cDNA to produce an amplicon of 152 bp. Real-time-PCR was performed in a C1000™ Thermal Cycler (BioRad CFX 96™ Real-Time System) according to the manufacturer's protocol. The final volume of each Real-time-PCR reaction was 20 μl, which contained 10 μl 2×SYBR Premix ExTaq (TaKaRa, Japan), 2.0 μl of 10-fold diluted cDNA template, 6 μl of PCR-grade water, and 1.0 μl of each 10 μM primer. PCR conditions were as follows: 95°C for 30 s, followed by 40 cycles of 95°C for 5 s and 60°C for 30 s. Each sample was run in triplicates and normalized to the selected control gene *β-*actin of *S. meridionalis*. The primers of *β-actin* (Table 1) were designed to produce an amplicon of 342 bp.

*Stra8* expression levels were calculated by the 2^−ΔΔCt^ Comparative CT method [28]. Mean and standard deviations were calculated from triplicate experiments, and presented as n-fold differences in expression relative to *β-actin*. Data was analyzed using the the CFX Manager™ software (version 1.0). Data is reported as mean ± standard error of mean (S.E.). The homogeneity of variance was confirmed and comparison between means was performed with a one-way ANOVA. Duncan's procedure was used for multiple comparisons between groups. Differences were regarded as significant when P<0.01. All statistical analyses were performed by STATISTICA 6.0.

### Hemotoxylin-eosin (H.E.) staining and *in situ* hybridization (ISH)

Gonads were fixed in Bouin’s solution, embedded in paraffin, and cross-sectioned at 5 μm. Sections were deparaffinized, hydrated, H.E. stained and mounted.

Gonads were fixed in 4% paraformaldehyde in 0.85× PBS (pH 7.4) at 4°C. After fixation, gonads were embedded in paraffin. Cross sections were cut at 5 μm. Probes of sense and anti-sense digoxigenin-labeled RNA strands were transcribed *in vitro* with an RNA labelling kit (Roche, Germany) from plasmid DNA containing ORF of the *Stra8*. The hybridization was carried out as follows: sections (5 μm) were deparaffinized, hydrated and treated with proteinase K (Amersco, USA.) and then hybridized with the sense or antisense DIG-labelled RNA probe at 60°C for 18-24h. The hybridization signals were then detected using alkaline phosphatase-conjugated anti-DIG antibody (Roche, Germany) and NBT as described previously [29].

### Stra8 recombinant protein expression, antibody production and Western blot analysis

An expression vector of Stra8 was constructed using pCold I. Recombinant *S. meridionalis* Stra8 with His-tag at its N-terminal was expressed in *E. coli* by IPTG induction until the absorbance value reached an OD_600_ of 0.5-0.6. His-Stra8 recombinant protein purified using Ni-NTA superflow cartridge (Qiagen) was used as the antigen to immunize rabbit (female, from Chongqing Medical University Animal Centre) three times at 15 day intervals with 25–30 μg antigen each time for the production of polyclonal antibodies. Ten days after the last immunization, rabbit blood was collected for ELISA evaluation. To confirm the polyclonal antibody specificity, total proteins extracted from both the fish gonads as well as the His-Stra8 recombinant proteins (both purified and unpurified) were separated on 12% SDS-PAGE (sodium dodecyl sulfate-polyacrylamide gel electrophoresis) gels. The separated proteins were transferred onto PVDF membranes and immunoblotted with the primary antibodies against Stra8 (Anti-Stra8, ×1000). After PBS washing, chemiluminescence was applied for the detection after incubation with secondary antibody conjugated with horseradish peroxidase. In order to enhance the signal of Stra8 protein from fish gonads, chemiluminescence was performed as described previously [5], and the signal was visualized on Fusion Fx (BioRad, USA) using super signal west picoluminol /Enhancer Solution and super signal west picostable peroxide Solution (Pierce, USA) as substrates after PBS washing.

### Immunohistochemistry (IHC)

After washing with 0.01 M PBS three times for 10 min per wash, sections were immersed in 0.01 M citric acid buffer (pH 6.0) containing 0.1% Tween 20, and autoclaved for 5 min. The sections were then treated in a blocking solution (Roche, China), incubated with Stra8 rabbit polyclonal antibody (1:1000) overnight at 4°C, and rinsed with 0.01 M PBS three times for 5 min per wash. Subsequently, the tissue sections were incubated with a second antibody (goat anti-rabbit IgG) conjugated with horseradish peroxidase (Bio-Rad) at 1:2000 for 30 min, and then rinsed with PBS three times for 5 min per wash. Immunoreactive signals were visualized using diaminobenzidine (Sigma) as the substrate. Sections were counterstained with hematoxylin. For the negative control, the primary antibody was replaced with normal rabbit serum.

## Competing interests

The authors declare that they have no competing interests.

## Authors’ contributions

RD performed the experiments on ISH, ICH and drafted the manuscript. SY did the work on H.E. staining and ISH. JJ cloned the *Stra8* cDNA and did the work on tissue distribution. TW and HS did the work on western blot. LZ did the work on bioinformatics’ analysis. YZ and DW designed and directed the study and write the manuscript. All authors read and approved the final manuscript.
